# Ultrasound Imaging in Dentistry: A Literature Overview

**DOI:** 10.3390/jimaging7110238

**Published:** 2021-11-14

**Authors:** Rodolfo Reda, Alessio Zanza, Andrea Cicconetti, Shilpa Bhandi, Gabriele Miccoli, Gianluca Gambarini, Dario Di Nardo

**Affiliations:** 1 Department of Oral and Maxillofacial Sciences, Sapienza University of Rome, 00161 Rome, Italy; rodolforeda17@gmail.com (R.R.); ale.zanza@gmail.com (A.Z.); andrea.cicconetti@uniroma1.it (A.C.); gianluca.gambarini@uniroma1.it (G.G.); dario.dinardo@uniroma1.it (D.D.N.); 2 Department of Restorative Dental Sciences, College of Dentistry, Jazan University, Jazan 45142, Saudi Arabia; shilpa.bhandi@gmail.com

**Keywords:** ultrasounds, dentistry, echography, ultrasonography

## Abstract

(1) Background: the frequency with which diagnostic tests are prescribed with exposure to ionizing radiation, a cause of biological damage, has been studied, and with much more attention, patients are subjected to these diagnostic tests for diagnosis and follow-up. This review aimed, given the recent developments of this technology, to evaluate the possible use of ultrasound in different branches of dentistry. The possibility of applying ionizing-radiation-free diagnostic exams in dentistry, overcoming the limits of this application, has led scientific research in this area to obtain interesting results that bode well for the future. (2) Methods: a search for articles on the application of ultrasounds in dentistry was performed using the PubMed electronic database. (3) Results: only 32 studies were included, and these clearly stated that this examination is widely usable and in great progress. (4) Conclusions: regarding the modern application techniques of this diagnostic test, it is essential to consider technological evolution as an objective to reduce the damage and side effects of necessary diagnostic tests. The use of ultrasound in dentistry can represent a valid radiation-free alternative, in certain contexts, to the other most used exams.

## 1. Introduction

In recent years, increasing attention has been paid to the frequency with which the patients are subjected to diagnostic exams that exploit ionizing radiation, a cause of biological damage, for diagnosis and follow-up [1,2]. Scientific progress has led in recent years to considerably lower the dose of radiation emitted by the latest-generation devices to obtain increasingly high-resolution diagnostic exams and applications in every branch of dentistry [3,4]. Moreover, the possibility of applying ionizing-radiation-free diagnostic exams in dentistry, overcoming the limits of this application, has led scientific research in this area to obtain interesting results that bode well for the future. Magnetic resonance imaging (MRI) and ultrasound imaging represent the most interesting evolution of this topic, as underlined by numerous evidence obtained in every branch of dentistry from the application of these diagnostics exams [5,6,7]. The main disadvantage of this examination remains the difficult visualization of tissues poor in water, which, however, has proven to be correctable by dedicated software, and can lead to excellent results. Patients suffering from claustrophobia, the presence of devices that prevent the examination from taking place, artifacts from materials and movements, the cost, the lack of availability, and the long examination time represent other disadvantages that will need to be improved in the future [6,7]. Examinations of this type, in addition to providing useful indications for diagnosis, can also be used with greater certainty as regards patient follow-up, being repeated at relatively short distances, without causing biological damage [8,9,10].

Differently than X-rays, sound waves can be represented as a mechanical longitudinal wave, which can manifest as particle displacement or pressure alterations. To understand the physics of ultrasound, and the possible application in dentistry, it is necessary to define the most important physical quantities that participate in the aforementioned mechanism: frequency, propagation speed, pulsed ultrasound, interaction with tissues, angle of incidence, and attenuation [11].

**Frequency**: This characteristic of ultrasonic waves is represented by cycles or pressure changes that occur in 1 s (Hertz). The aforementioned characteristic is determined exclusively by the sound source, and is not affected by the medium in which the wave is propagated. In this regard, it is fair to point out that ultrasound has an emission frequency greater than 20 kHz, at the upper limit of human hearing. Ultrasounds with frequencies up to 10 MHz are usually used in medical practice [5,11]. Furthermore, an important and current field of development of ultrasound imaging is represented by the application of high-frequency ultrasound (HFUS), which includes an ultrasound probe frequency of more than 10 MHz. HFUS has shorter wavelengths and is absorbed more easily, and is therefore not as penetrating. This feature makes it possible to apply it in the study of superficial structures, and hence its increasing application in the field of dermatology. This application feature is also interesting for applications in dentistry [11].

**Propagation speed**: This characteristic represents the speed at which ultrasound propagates through a medium; it is considered to be 1540 m/s for soft tissues. Unlike the frequency, this characteristic depends exclusively on the characteristics of the medium in which the wave propagates, density and rigidity above all.

**Pulsed ultrasound**: This represents an instrument that allows the emitting of short bursts of ultrasonic waves from a generator. For different clinical applications, different depths of resolution are needed, and pulsed rays (produced intermittently) are used. The duration of the pulse is about a millisecond.

**Interaction of ultrasound with tissues**: This feature describes what happens to an ultrasound beam that propagates through a medium. The reflection of the ray is called echo, and this is the fundamental property of this type of examination that allows the clinical evaluation of deep structures. The echoes’ generation and acquisition allow the evaluation of the depth of tissues at the level of their interface, allowing the analysis of the physical characteristics of different materials, studied as acoustic impedance. As long as the beam passes through media with the same acoustic impedance, it will not produce any reflection of the signal, and therefore no echo. It is important to underline that it is the difference between the acoustic impedances of neighboring tissues that determines the number of reflected echoes. The greater the echoes produced, the smaller the number of rays that make up the beam that crosses the second medium, and therefore the greater the intensity of the beam of rays that is reflected. In low-density tissue, the intensity of echoes produced at an interface between two layers is only a small percentage of the beam of rays. Precisely for this reason, if the interface is between two tissues with a large difference in densities, it will be impossible to read the areas of interest underlying this interface, and therefore the operator conducting the examination must avoid these areas, making this test sensitive to dependence on the operator who practices it, and requiring a rather long learning curve compared to other diagnostic exams [11].

**Angle of incidence**: The importance of this property is given by the fact that if the ultrasound beam hits the border obliquely, it is partially reflected and part of this echo is not received by the probe, making the interactions with the tissues more complex, and therefore, the clinical evaluation of the image produced. The process by which part of the beam will be deflected, dependent on the speed of the ultrasound at the sides of the interface, is called refraction. Snell’s law describes this phenomenon, allowing the calculation of the amount of deflection of the beam, relating the angle of refraction with the speed of the ultrasonic beam crossing that interface.

The first pulse generated and echoes produced at the interfaces are the most relevant characteristics [11]. Differential amplification can be used for the study of the weaker reflections that come from interfaces deep in the structures; typically, the pulses are a millisecond long [11].

In describing the reflection and refraction of the rays and fundamental elements of this type of examination so far, we have considered only stationary interfaces. If the interfaces move relative to the sound source, the frequency of the echo will be changed. As the sound source approaches, it will seem to have increased. This phenomenon is described in what is referred to as the Doppler effect [12]. In this technology, the transducer is stable, and the interfaces studied are often in motion. Depending on whether the movement of the interface is towards or away from the probe, the information received will be different. This application is extremely interesting in the diagnosis of blood vessels or vascular lesions [13]. The instrumentation currently in use allows the calculation of the flow in each point of the image, and to associate a color scale with the entire image that highlights the entire flow captured at that moment. Moreover, Doppler displays flux size. Power Doppler is more flux sensitive.

In this brief description of the physical characteristics of this equipment, the formation of artifacts remains to be evaluated, which may not recommend its application in some clinical uses. An important problem encountered in diagnostic tests is represented by the presence of artifacts, which are incorrect representations of the figures. These phenomena are often produced by physical characteristics that modify the representation of the image, as regards the use of ionizing radiation, magnetic waves, or ultrasound beams, which in some cases can even lead to diagnostic errors. To be able to evaluate them correctly, it is necessary to evaluate the ultrasound image production process:

- Sound waves move in straight lines.
- Reflections are generated from structures along the central axis of the beam.
- Amplitude of reflection corresponds to the reflector scattering strength.
- Sound moves at exactly 1540 m/s.
- Sound moves directly to the reflector and back.

Sometimes, it is not possible to say that the artifact occurred for these reasons. Certainly, most artifacts can lead to diagnostic errors, while others can represent an important clinical indication for making a diagnosis [11,13]. The main artifacts that can be encountered in the use of ultrasound are indicated and briefly summarized below:

- Reverberation: these artifacts manifest as multiple lines at the same distance to a ray line, generated by multiple sound echoes from the same interfaces.
- Ring down: these images are generated when small bodies produce the same resonance of ultrasound for the emitted sound. This sound is produced after the first reflection, and when it is received by the probe, the device interprets this signal as coming from deeper interfaces in the medium.
- Mirror images: sound can bounce off a strong, smooth reflector such as the diaphragm. The surface acts as mirror and reflects the pulse to another tissue interface, and from this image, it seems that the second interface is beyond the first.
- Reflections: a mechanism that can be superimposed on the mirror image, different in a peculiar way in how it appears, and is generated by different reflections, producing the effect that there are deeper structures than the one studied.
- Enhancement: this artifact is seen as an abnormally high brightness. This occurs when sound travels through a medium with an attenuation rate lower than surrounding tissue.
- Attenuation: the interfaces placed below media that reflect most of the ultrasounds, such as calcification, are represented as less echo-intense because the intensity is attenuated [11,13].

A described operation in broad lines of the device, and the physical properties that suggest the most suitable clinical uses in its application, increasingly greater in recent years, has been sought in every branch of dentistry.

## 2. Materials and Methods

A search for articles on the application of ultrasounds in dentistry was performed using the PubMed electronic database. A total of 428 articles were screened, and only 32 studies were included. According to the authors, only some articles about oral surgery, oral pathology, cranio-cervical surgery, gnathology, and orthodontics, which best represented the aim of this study, were selected. The only articles selected and not related to these branches of dentistry were considered only for the technical specifications and considerations on the functioning of ultrasound devices. All research articles concerning ultrasound that did not provide significant indications about the indication for the use of this technology, or for its improvement, were excluded from this study. Furthermore, the article that more than others represented the indication for the daily use of this technology was included in each branch of dentistry considered. Considering the scope of this article, all applications of ultrasound systems not related to echography were excluded.

## 3. Results

**Figure 1:**
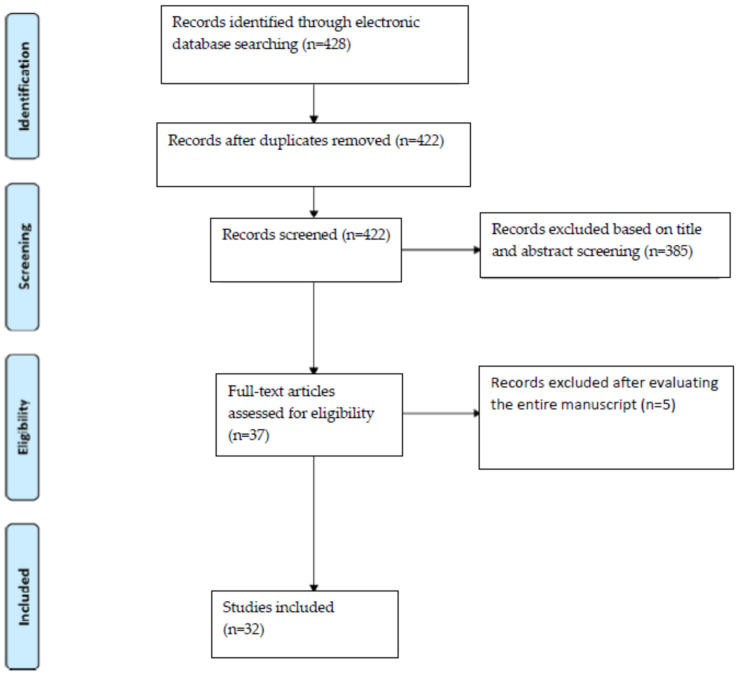
Search flow performed according to the PRISMA statement (Preferred Reporting Items for Systematic Reviews and Meta-Analyses) [14].

The articles taken into consideration indicated that this examination is widely usable and in great progress. All the articles considered for the study are indicated in Table 1. The research flow is also indicated in Figure 1. The number of articles concerning this technique, and their topicality, all very recent, represent a great invitation to continue the research on this radiation-free technique to allow a rapid and effective clinical application. The absence of ionizing radiation places great interest in the development of specific models for dental use, and great possibilities for improving its application in the coming years.

## 4. Discussion

The main application of ultrasound in dentistry in the past years has always been the diagnosis of pathologies of the major salivary glands, and sialolithiasis. Structural changes can be visualized as hyperechogenic and hypoechogenic areas, inhomogeneity, and altered echogenicity in general. A very interesting application is the study of Sjögren’s syndrome, as investigated by Jonsson et al. [15]. This examination, due to its reduced invasiveness and absence of radiation, and an investigation of the health of the salivary glands is indicated in any case in which problems of the major salivary glands may present with symptoms such as dry mouth, dysphagia and obstruction of duct, inflammation, severe dental caries, or swelling [16]. Moreover, an increasing amount of scientific evidence is being found in the study of periapical lesions, in the follow-up of their healing, and in the attempt to differentiate them in the different hypotheses of differential diagnosis. This examination represents the best diagnostic aid for these diagnoses, and has represented it for years as regards the study of the superficial structures of the head and neck area, such as the lymph nodes [17]. Moreover, the approach to the condition of muscular health becomes increasingly central in establishing the correct balance even in orthodontic–gnathological treatments, the planning of which is increasingly facilitated by current 3D cephalometry software, MRI, and auxiliary study examinations of the occlusal balance [4,7,18].

Often in the evaluation of the health of the temporomandibular joint, an MRI is prescribed that can hardly be carried out in the same diagnostic center. From this point of view, the ultrasound examination could represent valid help in the study of the joint and the state of health of the articular disc or its possible displacement [19]. In addition to the evaluation of the joint soft tissues, this examination allows a careful study of the superficial soft tissues, and therefore of the oral mucosa. As pointed out by Zompas et al., the measurement of thickness of the maxillary attached gingiva is extremely useful when a connective tissue removal from the palate is assumed for a free graft [20]. Evidence of the use of MRI for this purpose is also reported in the literature; however, it is unfortunately difficult to perform [7]. This application of ultrasound could be extremely useful in planning periodontal surgeries. The possibility of studying the oral mucosa by ultrasound has been extensively investigated in the research of Izzetti et al., who reported extremely interesting results: for all the sites analyzed in this study, ultrahigh-frequency ultrasound (UHFUS) biomarkers were characterized, and information on typical aspects of the oral mucosa was retrieved. These findings encourage the use of this method in the study of surface soft tissues in all their layers [21]. The possibility of adequately analyzing the soft tissues and their layers allows practitioners to reach even districts that are difficult to explore intraorally, and to evaluate their state of health or their possible compromise in inflammatory processes through a radiation-free examination [22].

Similar evaluations can also be conducted with regard to implant surgery and peri-implant health, while always remembering that this follow-up does not expose the patient to risk of exposure to ionizing radiation, and therefore is free from biological damage [23]. In an interesting article by Bohner et al., there was no statistical difference among the different groups for the measurements of the thickness of the buccal bone surrounding dental implants, whether measured with ultrasound or with an optical microscope [24]. With the ex vivo limitations of this study, it was, however, possible to draw an interesting starting point in the study of peri-implant hard tissues as well. To confirm the accuracy of the results obtained previously, the same research group simulated peri-implant bone defects on porcine bone, and after performing CBCT, evaluated the same defects with the ultrasound method, obtaining extremely interesting results: no statistically significant differences in the measurements of width and height were found [25]. The same evaluations can be carried out on the soft tissues peri-implant, recalling the previously cited studies on the analysis of the oral mucosa and its thickness, especially palatal, for the planning of periodontal surgery [20,21,26]. In the study by Culijat et al., ultrasound was used to accurately detect, locate, and measure dental implant fixtures, and measure the thickness of the overlying soft tissue in an ex vivo environment [27]. The results of a study by Vayron R. et al. showed that the ultrasonic response of a dental implant varied significantly as a function of healing time, which paved the way for the development of a new quantitative ultrasound (QUS) method in oral implantology [28,29,30,31]. An important application of this technology can be considered extremely useful for the evaluation of intraosseous lesions, their diagnosis and differential diagnosis, and their follow-up after being treated, while always remembering the absence of ionizing radiation [32,33]. Ultrasonography has been used effectively for the diagnosis of inflammatory lesions, cysts, nonodontogenic and odontogenic tumors, and arteriovenous malformations, and for the differential diagnosis of lesions of endodontic origin. It may be a viable adjunct to other special tests for the diagnosis of intraosseous lesions of the jaw [34]. A study by Arslan et al. found ultrasonography to be an alternative method to digital radiographic techniques in the diagnosis of anterior teeth with periapical lesions [35]. Regarding the differential diagnosis between different bone lesions, the results of a study by Sönmez et al. suggested good agreement between ultrasound and histopathology. No statistically significant differences were found among periapical radiography, CBCT, and ultrasound in the measurements of lesions [36,37]. The potential of this examination has been well defined and underlined by previous articles, and it certainly represents an interesting evolution in this application [38,39]. In the logic of a radiation-free future, especially in the follow-up of bone surgery (in this case root-end surgery), the possibility of using an examination of this type, especially in the anterior maxilla and mandible, where the dimensions of the probe are not a limitation, it proved to be a valid alternative, with full respect to patient safety, representing the first and true guide in planning any medical and surgical treatment [40,41,42].

Dento-periodontal tissues were studied by Nguyen et al. with a phased array system with a 128-element array transducer. The high-resolution ultrasonographs clearly showed the cross-sectional morphological images of the hard and soft tissues of the pig’s jaw. The alveolar crest level, the location of cement–enamel junction, and the thickness of alveolar crest were measured from the images and compared favorably with those from the cone-beam computed tomography, with less than a 10% difference. This preliminary and fundamental study reinforced the conclusions from previous studies that ultrasonography has great potential to become a noninvasive diagnostic imaging tool for quantitative assessment of periodontal structures [32,43,44].

An important application of ultrasound in medicine is represented by the study of blood vessels, and in fact, when applied to dentistry, turns out to be a very important examination in the evaluation of those needing oral surgery of soft tissues in the vicinity of large blood vessels, or of vascular lesions to understand their nature and the flow within them [45]. For years, the only possibility to study the positioning of blood vessels was to evaluate them with CT or CBCT, with little definition. Only in recent years, the reduction of the dosage of these tests, but with radiation always present, and with the biological damage that it entails, and the increase in the definition of these diagnostics exams has led to an important improvement in the evaluation of the position of blood vessels in hard tissues hard, but not very effectively for soft tissues, where instead this examination guarantees excellent intraoperative results, with zero biological risk [45,46].

As we have seen previously with regard to gnathology and orthodontics, the application of this exam for the study of soft tissues opens up the possibility of monitoring the muscular state after prosthetic or implant-prosthetic rehabilitation, an extremely important relationship and always to be taken into consideration for stability of the occlusion and follow-up of prosthetic restorations in the presence of parafunctions [47,48].

As we explained in the introduction of this article, as regards the operation of this imaging technique, there must not be an interface with extremely different densities at the surface level (almost always air) to avoid image distortion or its lack of display. In this regard, research is already leading to colloids with excellent affinity with the tissues of the oral cavity for study with intra-oral ultrasound [49].

Kim et al. evaluated the sizes of caries with this method, in particular with high-frequency ultrasound (HFUS), comparing them with data obtained by CT [50]. The extremely promising results allow us to consider this possibility, from the moment in which probes suitable for this use will be available, very valid for the study of caries dimensions. In fact, having knowledge of the exact size of the caries that one must face allows us to establish exactly the type of restoration most suitable for the rehabilitation of the specific case [51]. Furthermore, being able to immediately know the dimensions of the caries that one is to restore can allow one to devise a specific endodontic access, guided by the caries, to maintain as much dental substance as possible, and exploit the physical-mechanical properties of the new alloys and new rotating instruments. Ni-Ti is on the market [52,53,54,55]. Up to now, this evaluation could only be done with certainty from the moment in which the cavity was made, the caries cleaned, and the possibility of guided access from the cavity was evaluated [51,52,53,54,55].

It is necessary to conduct comparative studies of the various systems used in oral ultrasound imaging to obtain a consensus or guidelines to provide clinicians with the decision-making criteria in the choice of a type of device. All the studied characteristics are indicated in Table 2.

From what emerged from this first analysis of the clinical use of ultrasound, the most used transducer was the linear one, and with a variable frequency from 5 to 70 MHz, frequently in the range from 10 MHz to 25 MHz. The most used probe in these studies was a linear probe, often used in vascular imaging. What makes it the ideal probe for vascular imaging is its possible central positioning, which is certainly more complicated, however, in the oral cavity.

The last consideration to be made regards the preparation of the students, in their university and preclinical study programs, for this modern imaging technique, which must increasingly, like magnetic resonance imaging (MRI), be considered a valid alternative without ionizing radiation, in the diagnosis and follow-up of various conditions of the oral cavity [6,7,52,56].

## 5. Conclusions

In light of the results obtained in the various fields of study of the modern application techniques of this diagnostic test, it is essential to consider technological evolution as an objective to reduce the damage and the side effects of necessary diagnostic tests, which are increasingly prescribed for diagnosis, follow-up, and defensive medicine. The use of ultrasound in dentistry, if the investments allow the development of probes and instruments suitable for the oral cavity, will prove to be an important aid, similar to magnetic resonance, and which, despite the limitations of these tests, can represent a valid alternative, in certain contexts, that is always radiation-free.

## Figures and Tables

**Table 1:** Articles included in this study.

Title	Authors	Year
Major Salivary Gland Ultrasonography in the Diagnosis of Sjögren’s Syndrome: A Place in the Diagnostic Criteria?	Jonsson MV, Baldini C.	2016
Diagnostic imaging in salivary gland disease.	Afzelius P, Nielsen MY, Ewertsen C, Bloch KP.	2016
Electromyographic, Ultrasonographic, and Ultrasound Elastographic Evaluation of the Masseter Muscle in Class III Patients Before and After Orthognathic Surgery.	Sunal Akturk E, Eren H, Gorurgoz C, Orhan K, Karasu HA, Akat B, Toygar Memikoglu TU.	2020
Ultrasonography for diagnosis of peri-implant diseases and conditions: a detailed scanning protocol and case demonstration.	Chan HL, Kripfgans OD.	2020
Diagnostic value of ultrasonography for the detection of disc displacements in the temporomandibular joint: a systematic review and meta-analysis.	Su N, van Wijk AJ, Visscher CM, Lobbezoo F, van der Heijden GJMG.	2018
Ultrasound Assessment of Bone Healing after Root-end Surgery: Echoes Back to Patient’s Safety.	Curvers F, Meschi N, Vanhoenacker A, Strijbos O, Van Mierlo M, Lambrechts P.	2018
The Intraoral Ultrasonography in Dentistry.	Caglayan F, Bayrakdar IS.	2018
Recent advances of ultrasound imaging in dentistry—A review of the literature.	Marotti J, Heger S, Tinschert J, Tortamano P, Chuembou F, Radermacher K, Wolfart S.	2013
Ultrasound in Dentistry: Toward a Future of Radiation-Free Imaging.	Demirturk Kocasarac H, Angelopoulos C.	2018
Ultrasound Imaging versus Radiographs in Differentiating Periapical Lesions: A Systematic Review.	Patil S, Alkahtani A, Bhandi S, Mashyakhy M, Alvarez M, Alroomy R, Hendi A, Varadarajan S, Reda R, Raj AT, Testarelli L.	2021
Assessment of Buccal Bone Surrounding Dental Implants Using a High-Frequency Ultrasound Scanner.	Bohner L, Habor D, Tortamano P, Radermacher K, Wolfart S, Marotti J.	2019
Polyacrylamide/Alginate double-network tough hydrogels for intraoral ultrasound imaging.	Yi J, Nguyen KT, Wang W, Yang W, Pan M, Lou E, Major PW, Le LH, Zeng H.	2020
Ultrasonography in the diagnosis of bone lesions of the jaws: a systematic review.	Musu D, Rossi-Fedele G, Campisi G, Cotti E.	2016
Diagnostic accuracy of panoramic radiography and ultrasonography in detecting periapical lesions using periapical radiography as a gold standard.	Arslan ZB, Demir H, Berker Yıldız D, Yaşar F.	2020
Ultrasonic Measurement of Lingual Artery and Its Application for Midline Glossectomy.	Liu C, Qin J, Xing D, Lu H, Yue R, Li S, Wu D.	2020
Ultrasound Examination to Visualize and Trace Sinus Tracts of Endodontic Origin.	Cotti E, Musu D, Goddi A, Dettori C, Campisi G, Shemesh H.	2019
Ultra-High Frequency Ultrasound, A Promising Diagnostic Technique: Review of the Literature and Single-Center Experience.	Izzetti R, Vitali S, Aringhieri G, Nisi M, Oranges T, Dini V, Ferro F, Baldini C, Romanelli M, Caramella D, Gabriele M.	2021
Discovering a new anatomy: exploration of oral mucosa with ultra-high frequency ultrasound.	Izzetti R, Vitali S, Aringhieri G, Oranges T, Dini V, Nisi M, Graziani F, Gabriele M, Caramella D.	2020
Accuracy of High-Frequency Ultrasound Scanner in Detecting Peri-implant Bone Defects.	Bohner L, Habor D, Gremse F, Tortamano P, Wolfart S, Marotti J.	2019
The Role of Ultrasound and Shear-Wave Elastography in Evaluation of Cervical Lymph Nodes.	Heřman J, Sedláčková Z, Fürst T, Vachutka J, Salzman R, Vomáčka J, Heřman M.	2019
Versatility of high resolution ultrasonography in the assessment of granulomas and radicular cysts: a comparative in vivo study.	Sönmez G, Kamburoğlu K, Yılmaz F, Koç C, Barış E, Tüzüner A.	2019
Ultrasonography for noninvasive and real-time evaluation of peri-implant tissue dimensions.	Chan HL, Sinjab K, Li J, Chen Z, Wang HL, Kripfgans OD.	2018
The effectiveness of ultrasound examination to assess the healing process of bone lesions of the jaws: a systematic review	Davide M, Hagay S, Michela B, Claudia D, Elisabetta C.	2020
Integration of ultrasound imaging into pre-clinical dental education.	Kondrashova T, De Wan D, Briones MU, Kondrashov P.	2017
High-Frequency Ultrasound Imaging for Examination of Early Dental Caries.	Kim J, Shin TJ, Kong HJ, Hwang JY, Hyun HK.	2019
Mastication improvement after partial implant-supported prosthesis use.	Gonçalves TM, Campos CH, Gonçalves GM, de Moraes M, Rodrigues Garcia RC.	2013
Updates on ultrasound research in implant dentistry: a systematic review of potential clinical indications.	Bhaskar V, Chan HL, MacEachern M, Kripfgans OD.	2018
Utility of Transfacial Dental Ultrasonography in Evaluation of Cystic Jaw Lesions.	Gad K, Ellabban M, Sciubba J.	2018
Ultrasound imaging of dental implants.	Culjat MO, Choi M, Singh RS, White SN.	2012
High-Resolution Ultrasonic Imaging of Dento-Periodontal Tissues Using a Multi-Element Phased Array System.	Nguyen KT, Le LH, Kaipatur NR, Zheng R, Lou EH, Major PW.	2016
Ultrasound real-time imaging in the differential diagnosis of periapical lesions.	Prince CN, Annapurna CS, Sivaraj S, Ali IM.	2012
The Use of High Frequency Ultrasound in the Measurement of Thickness of the Maxillary Attached Gingiva.	Tzoumpas M, Mohr B, Kurtulus-Waschulewski I, Wahl G.	2015

**Table 2:** Technical characteristics.

Title	Year	Types of Transducers	Range of Frequencies	Advantages/Disadvantages of the Different Ultrasound Systems
Major Salivary Gland Ultrasonography in the Diagnosis of Sjögren’s Syndrome: A Place in the Diagnostic Criteria?	2016			Being user-friendly, rapidly performed, repeatable, noninvasive, and nonradiating, SG-US has emerged as a promising diagnostic and prognostic tool.
Diagnostic imaging in salivary gland disease	2016		7–15 MHz	It can be used for image guided biopsies, and can be performed in the emergency setting. Ultrasound has limitations in evaluating structures behind bone and the deep parts of the parotid gland.
Electromyographic, Ultrasonographic, and Ultrasound Elastographic Evaluation of the Masseter Muscle in Class III Patients Before and After Orthognathic Surgery	2020	Convex transducers	3–5 MHz	Muscle length, thickness, cross-sectional area, and volume measurements can be obtained with ultrasound imaging.
Ultrasonography for diagnosis of peri-implant diseases and conditions: a detailed scanning pro-tocol and case demonstration	2020	Toothbrush-sized (~30 mm × 18 mm × 12 mm) probe	25 MHz	It displays images of peri-implant tissues of various health conditions in live humans.
Diagnostic value of ultrasonography for the detection of disc displacements in the temporomandibular joint: a systematic review and meta-analysis	2018			Ultrasound can be considered as a relevant imaging tool to supplement clinical examination in patients with suspected disc displacement in selected cases. Combined static and dynamic examinations using high-resolution ultrasound should be preferred.
Ultrasound Assessment of Bone Healing after Root-end Surgery: Echoes Back to Patient’s Safety	2018	Linear ultrasonic probe operating	12 MHz	It can detect initial bone healing processes.
The Intraoral Ultrasonography in Dentistry	2016		2.5–10 MHz, up to 40 MHz	Intraoral ultrasound examination is limited to the anterior aspects of the jaws, as the presently available probes are not ideal for use in the posterior jaws in areas of thick cortical plates.
Recent advances of ultrasound imaging in dentistry—a review of the literature	2013		2–20 MHz	Ultrasonography may provide a significant benefit to patients by allowing early detection of tooth lesions and defects, measurement of mucosa and gingival thickness, dental implant locations, and dental scanning.
Ultrasound in Dentistry: Toward a Future of Radi-ation-Free Imaging	2018		3–12 MHz	It provides real-time and simultaneous imaging of both hard and soft tissues.
Ultrasound Imaging versus Radiographs in Differentiating Periapical Lesions: A Systematic Review	2021		6–12 MHz	Within the limitations of the studies included, this review indicates that it provides better diagnostic accuracy for differentiating endodontic lesions compared to radiographic imaging.
Assessment of Buccal Bone Surrounding Dental Implants Using a High-Frequency Ultrasound Scanner	2019	Transducer spherically focused with an aperture of 6 mm and focus of 13.2 mm	28 MHz	High-frequency ultrasound was able to measure buccal bone dimensions surrounding dental implants with a trueness similar to that of cone-beam computed tomography.
Polyacrylamide/alginate double-network tough hydrogels for intraoral ultrasound imaging	2020	6.35 mm diameter unfocused transducers	20 MHz	PAM/alginate tough hydrogels were explored as potential couplants for intraoral ultrasound imaging by a comprehensive comparison of their physical, mechanical, frictional, and ultrasound properties, as well as biocompatibility with the commercial couplant.
Ultrasonography in the diagnosis of bone lesions of the jaws: a systematic review	2016			The results demonstrated the value of ultrasonography for the evaluation of the nature of intra-osseous lesions in the jaws.
Diagnostic accuracy of panoramic radiography and ultrasonography in detecting periapical lesions using periapical radiography as a gold standard	2020	Linear ultrasonic probe	7–10 MHz	These results showed that although the ultrasound has a higher value than the panoramic, the two techniques have similar diagnostic accuracy values, and there is no significant difference between the two techniques in the detection of periapical lesions.
Ultrasonic Measurement of Lingual Artery and Its Application for Midline Glossectomy	2020			In conclusion, preoperative US can show the course of the lingual artery clearly for preoperative planning.
Ultrasound Examination to Visualize and Trace Sinus Tracts of Endodontic Origin	2019	Linear and multifrequency probes	7–12 MHz	Ultrasound real-time examination can be successfully used to detect the STs of endodontic origin and to trace their route of drainage from the periapical lesion to the opening within the oral mucosa or the skin.
Ultra-High Frequency Ultrasound, A Promising Diagnostic Technique: Review of the Literature and Single-Center Experience	2021		30–70 MHz	The literature on UHFUS is still evolving, but ultrahigh frequencies seem to be the answer to several clinical problems related to the high-resolution investigation of both normal anatomy and disease processes.
Discovering a new anatomy: exploration of oral mucosa with ultra-high frequency ultrasound	2020		70 MHz	It is considered to be a diagnostic support in the management of oral soft tissue lesions, in terms of diagnosis, surgical procedure, postoperative discomfort reduction, and prevention/early detection of malignant transformation.
Accuracy of High-Frequency Ultrasound Scanner in Detecting Peri-implant Bone Defects	2019	Custom spherically focused transducer with an aperture of 4 mm	42 MHz	High-frequency ultrasound in association with the a priori information technique was accurate in measuring the width of peri-implant defects.
The Role of Ultrasound and Shear-Wave Elastography in Evaluation of Cervical Lymph Nodes	2019	Linear probe	4–15 MHz	Good results in discriminating benign from malignant cervical lymph nodes.
Versatility of high resolution ultrasonography in the assessment of granulomas and radicular cysts: a comparative in vivo study	2019	(1) Linear (2) Hockey probes	(1) 9 MHz (2) 15 MHz	It provides useful information for the diagnosis and assessment of granulomas and radicular cysts.
Ultrasonography for noninvasive and real-time evaluation of peri-implant tissue dimensions	2018		25 MHz	It could become a valuable method to evaluate peri-implant tissue biotype and peri-implant diseases.
The effectiveness of ultrasound examination to assess the healing process of bone lesions of the jaws: a systematic review	2020	Mainly linear	5–12 MHz	The USE implemented with CPD is an advanced imaging technique feasible for monitoring the early and long-term response of intra-osseous jaw lesions in both surgical and nonsurgical treatments.
Integration of ultrasound imaging into pre-clinical dentaleducation	2017			Results of the current study suggested that ultrasound could be integrated into dental education.
High-Frequency Ultrasound Imaging for Examination of Early Dental Caries	2018	Press-focused HFUS transducer	40 MHz	The invasion depths of WSLs obtained with HFUS images had good agreement with those of WSLs obtained with the micro-CT images within the limits of the study.
Mastication Improvement After Partial Implant-Supported Prosthesis Use	2013	Linear probe	7–18 MHz	The IRDPs and IFDPs significantly increased MBF and FCI, with the magnitude of the masticatory improvements closely related to prosthesis type.
Updates on Ultrasound Research in Implant Dentistry: A Systematic Review ofPotential Clinical Indications	2018			Limitations of ultrasound include the need of a medium for sound conduction, inability to penetrate into bone, and narrow field of view. Acoustic gel is needed.
Utility of Transfacial Dental Ultrasonography in Evaluation of Cystic Jaw Lesions	2018	Linear transducer	7–12 MHz	On transfacial dental US supplemented by a Doppler study with either a power or color display, vascular flow could be enhanced, and can be determinant in differential diagnosis.
Ultrasound imaging of dental implants	2012		16 Mhz	This experiment demonstrated that ultrasonography could be used to measure tissue depth over acoustically diffuse cancellous bone before implant placement, and to locate and measure soft tissue thickness over submerged implants.
High-Resolution Ultrasonic Imaging of Dento-Periodontal Tissues Using a Multi-Element Phased Array System	2016	Broadband array transducer	8–40 MHz	High-quality ultrasound images of the tooth and the surrounding periodontium.
Ultrasound imaging in the diagnosis of periapical lesions	2012	Linear transducer	7–11 MHz	With its potential usefulness to differentiate the periapical lesions, ultrasonography can be considered as a better imaging modality with improved efficacy when compared to conventional radiography.
The Use of High Frequency Ultrasound in the Measurement of Thickness of the Maxillary Attached Gingiva	2015	Linear probe	20 MHz	It has better characteristics, with the same results compared to a trans-mucosal probing.
